# A resource-efficient framework for plant disease classification: integrating reduced-order modeling with treatment-based label engineering

**DOI:** 10.3389/fpls.2026.1766704

**Published:** 2026-05-04

**Authors:** Youssef Natij, Hajar El Karch, Abdelmounaim Belaaribi, Oumaima Lanaya, Meriem El Atik, Ayyad Maafiri, Abdelkader Mezouari

**Affiliations:** 1 Scientific Research and Innovation Laboratory, Higher School of Technology, Ibn Tofail University, Kénitra, Morocco; 2 Laboratory of Advanced Systems Engineering, National School of Applied Sciences, Ibn Tofail University, Kénitra, Morocco; 3 Laboratory of Modeling and Combinatorics (LMC), Polydisciplinary Faculty of Safi, Cadi Ayyad University, Safi, Morocco

**Keywords:** computational efficiency, label engineering, plant disease classification, principal component analysis (PCA), reduced-order modelling, resource-efficient, YOLOv8

## Abstract

Plant disease diagnosis in field settings is challenged by subtle symptomology, high inter-class visual similarity, and class imbalance, making automated detection particularly difficult. While deep learning models achieve high accuracy, traditional architectures impose prohibitive computational costs that hinder deployment on resource-constrained hardware. This paper proposes a novel Reduced Order Modelling (ROM) framework integrating a YOLOv8m backbone for spatially sensitive feature extraction, PCA-based compression to isolate the most discriminative features, and classical classification. A treatment-based label engineering approach was applied to consolidate the PlantWildV2 dataset from 115 to 11 agronomically relevant classes. Experimental results showed that a highly compressed feature space acts as a natural regularizer, with accuracy peaking at 100 principal components and declining beyond that threshold. The tuned SVC classifier achieved a test accuracy of 87.52% and a macro F1-score of 0.882, outperforming all other classifiers evaluated. The proposed ROM framework surpassed EfficientNet-B0 in accuracy (87.52% vs. 82.50%) while reducing training time from 5.8 hours on GPU to 30.8 seconds on CPU, a 670-fold efficiency gain, demonstrating the viability of Reduced Order Modelling for plant disease detection on low-resource hardware.

## 1 Introduction

The world of agriculture, being so essential to our food supplies, is increasingly turning to artificial intelligence to secure yields and improve disease management (Tilman et al., 2011; Food and Agriculture Organization of the United Nations, 2021). Plant diseases are always a threat, with global crop losses exceeding $220 billion annually (Strange and Scott, 2005; Oerke, 2006; Savary et al., 2019). The conventional way of diagnosing plant diseases is through visual examination, but this is not only labor-intensive but also difficult to automate on a large scale due to the vast space that farms cover (Agrios, 2005; Barbedo, 2016). Luckily, with recent advancements in Deep Learning (DL), particularly Convolutional Neural Networks (CNNs) (LeCun et al., 1998; Krizhevsky et al., 2012; Schmidhuber, 2015), automatic plant disease detection using leaf images has been achieved with considerable success (Mohanty et al., 2016; Ferentinos, 2018; Too et al., 2019; Hassan et al., 2021; Lu et al., 2021).

However, when such models are tested with images collected in the field, their accuracy is significantly low (Barbedo, 2019; Liu and Wang, 2021; Xu et al., 2023; Richter and Kim, 2025). The images collected in the field are not as clean as those collected in a controlled environment, as there are not enough contrasts to distinguish healthy leaves from diseased ones, as well as not enough clarity to distinguish one type of disease from another (Barbedo, 2016; Albattah et al., 2022; Noyan, 2022). The problem is compounded by the similarities that exist between different kinds of plant diseases, as well as the dissimilarities that exist within one category of plant diseases, as seen in PlantWildV2 (Boulent et al., 2019; Wu et al., 2023; Wei et al., 2024). There is also another problem that is common in all agricultural datasets, known as class imbalance (He and Garcia, 2009; Jafar et al., 2024).

State-of-the-art DL models also demand substantial computation. Architectures such as VGG (Simonyan and Zisserman, 2014), ResNet (He et al., 2016), DenseNet (Huang et al., 2017), and EfficientNet (Tan and Le, 2019) contain millions of parameters and require high-end GPU hardware for training and inference (Alzubaidi et al., 2021; C and Murugavalli, 2022). This computational burden increases energy usage and latency and limits deployment on Edge AI devices where timely, on-field diagnosis is needed (Kamilaris and Prenafeta-Boldu, 2018; Liakos et al., 2018). Resource constraints, especially in smallholder agricultural settings in developing economies, remain a significant barrier to the adoption of DL-based diagnostic tools (Ngugi et al., 2021; Demilie, 2024).

To address both performance and computational efficiency, we investigate a hybrid DL-ML architecture that decouples feature extraction from classification. Such hybrid systems leverage robust deep representations while using lightweight classical machine learning for downstream prediction (Sharif Razavian et al., 2014; Islam et al., 2022; Demilie, 2024). The effectiveness of pre-trained CNN features for transfer learning is well established (Sharif Razavian et al., 2014; Yosinski et al., 2014), and recent hybrid approaches combining deep features with classical classifiers such as SVMs have shown promise in plant pathology (Bedi and Gole, 2021; Sahu and Pandey, 2023; Sharma et al., 2024). We propose, to our knowledge, the first use of a YOLOv8m backbone (Redmon et al., 2016; Jocher et al., 2023) as a feature extractor for plant disease classification. Our hypothesis is that the YOLOv8 backbone, optimized for object localization, yields spatially richer representations of localized disease symptoms (e.g., spots, lesions, necrotic regions) than standard classification backbones.

This work makes three contributions: (1) it demonstrates the efficacy of the YOLOv8m backbone as a feature generator for challenging, in-the-wild plant disease recognition; (2) it in- troduces a treatment-based label engineering strategy that consolidates 115 fine-grained classes into 11 actionable categories, improving accuracy and generalization; and (3) it identifies an effective pipeline (YOLOv8 features + PCA + tuned SVC) that achieves high accuracy with low computational overhead, supporting future implementation in resource-constrained envi- ronments.

## 2 Related work

The development in automated plant disease classification has been rapidly advancing with improvements in computer vision techniques (Dhaka et al., 2021; Shoaib et al., 2023). The review is based on recent studies that address the problems associated with deep learning in agriculture. The review is categorized into four main aspects: using deep learning models in plant disease identification, using deep learning models on limited resources, solving data-related problems, and using hybrid models in plant disease classification.

### 2.1 Deep learning for plant disease recognition

Convolutional Neural Networks (CNNs) are widely used in plant disease classification using images (Liu and Wang, 2021; Lu et al., 2021). Recent studies by Mohanty et al (Mohanty et al., 2016 ). and Ferentinos (2018) were landmark studies that showed that deep learning models such as VGG, ResNet, and GoogLeNet could achieve very high accuracy (>99%) on the PlantVillage dataset (Hughes and Salathe, 2015). Recent studies have shown improvements on this using transfer learning and fine-tuning (Too et al., 2019; Hassan et al., 2021). Recent reviews showed that CNNs were being widely used in various crops (Kamilaris and Prenafeta-Boldu, 2018; Boulent et al., 2019; Ngugi et al., 2021).

There is now a growing concern that deep learning models are not performing as well in the field as they do in the lab (Barbedo, 2016; Barbedo, 2019). Recent studies are trying to solve this problem. Salman et al (Salman et al., 2025 ). proposed a Vision Transformer (ViT) (Dosovitskiy et al., 2021) with a Mixture of Experts (MoE) to solve the generalization gap problem. Wu et al (Wu et al., 2023 ). proposed an unsupervised domain adaptation method called MSUN to solve this problem. Detection-based models are also being widely used. Pan et al (Pan et al., 2023 ). proposed Xoo-YOLO, a YOLOv8 variant that is widely used in UAV- based bacterial blight detection. Li et al (Li et al., 2022 ). proposed YOLOv5 architectures that were widely used in multi-scale disease recognition.

### 2.2 Resource-constrained and edge AI

Even with the improved accuracy, the existing deep learning models are still not feasible due to the high resources required by the existing models (Demilie, 2024; Jafar et al., 2024). Keeping this in mind, the more recent literature is focusing on developing lighter models or compressing the existing models. Nyakuri et al (Nyakuri et al., 2025), for instance, proposed the Tiny-LiteNet CNN-based model, which can run on the Raspberry Pi 5 device to detect pest and disease occurrences in real-time. Bhavani and Chalapathi (2026) proposed the lightweight SSD framework to detect potato diseases in real-time. They also highlighted the need to reduce the computational costs of the models without sacrificing the accuracy at the lesion level. Bhagat et al (Bhagat et al., 2024 ). proposed the lighter deep learning framework to detect the diseases in pigeon pea crops in real-time, while Ullah et al.

(Ullah et al., 2023) combined EfficientNet and MobileNet to detect the diseases in tomatoes more efficiently. Our work follows the same direction but takes a different path by using the frozen high-capacity YOLOv8 as the backbone and pushing the dimension reduction as hard as possible using PCA.

### 2.3 Data challenges and label engineering

The quality and representativeness of the datasets are still major limitations in the field of deep learning-based crop disease detection (Barbedo, 2016; Xu et al., 2023). Richter and Kim (2025) recently proposed the benchmarking of the transfer learning-based models on 18 public datasets and highlighted the bias and lack of real-world representativeness of the datasets as major limitations of the existing literature. Noyan (2022) also highlighted the background bias in the PlantVillage dataset, where the accuracy of the model using just eight background pixels was found to be as high as 49%. This highlights the possibility that the reported accuracy gains are due to background correlations rather than the actual presence of the disease. Keeping this in mind, the more recent literature is focusing on developing faster ways to generate the datasets using zero-shot detection and segmentation using tools such as Grounding DINO and SAM (Mullins et al., 2024; Mullins et al., 2025). More recent literature is also focusing on the dataset level by using techniques such as multi-output learning (Fenu and Malloci, 2021) and cross-crop generalization (Bouacida et al., 2024) to improve the transferability of the models. In this work, we are addressing the data quality by using label engineering techniques by combining the taxonomic classes with the treatment-based classes to reduce the ambiguity between the classes and align the output with the practical recommendations.

### 2.4 Hybrid pipelines and our contribution in context

Several recent studies have combined deep feature extraction with classical machine learning classifiers for plant disease tasks. Sharma et al (Sharma et al., 2024 ). proposed CSXAI, a CNN-SVM hybrid with explainable AI for crop disease classification. Sahu and Pandey (2023) developed a hybrid multi- class SVM with spatial fuzzy C-Means for leaf disease detection. El Akhal et al (El Akhal et al., 2023 ). introduced a deep hybrid model for olive leaf diseases, while Islam et al (Islam et al., 2022 ). combined attention-based di- lated convolution features with logistic regression for tomato disease classification. These works establish the viability of hybrid DL-ML pipelines; however, they rely on classification-oriented backbones.

Recent studies have explored end-to-end fine-tuning of YOLOv8 for plant disease tasks (Miao et al., 2024), but the use of YOLOv8 backbone features in a hybrid classification pipeline has not been systematically examined. We address this gap. Building on insights from our earlier review (Natij et al., 2024) and on dimensionality reduction techniques developed in prior work on face recognition (Maafiri and Chougdali, 2021; Maafiri et al., 2021), we hypothesize that feature hierarchies from object detection models, optimized for spa- tial localization, better capture localized disease patterns than classification-only architectures. We evaluate YOLOv8-derived features for downstream classification with Reduced-Order Mod- elling (Jolliffe, 2002; Halko et al., 2011; Benner et al., 2017), contributing evidence on repurposing modern detection backbones for transfer learning in challenging agricultural domains.

## 3 Methodology

**Figure 1:**
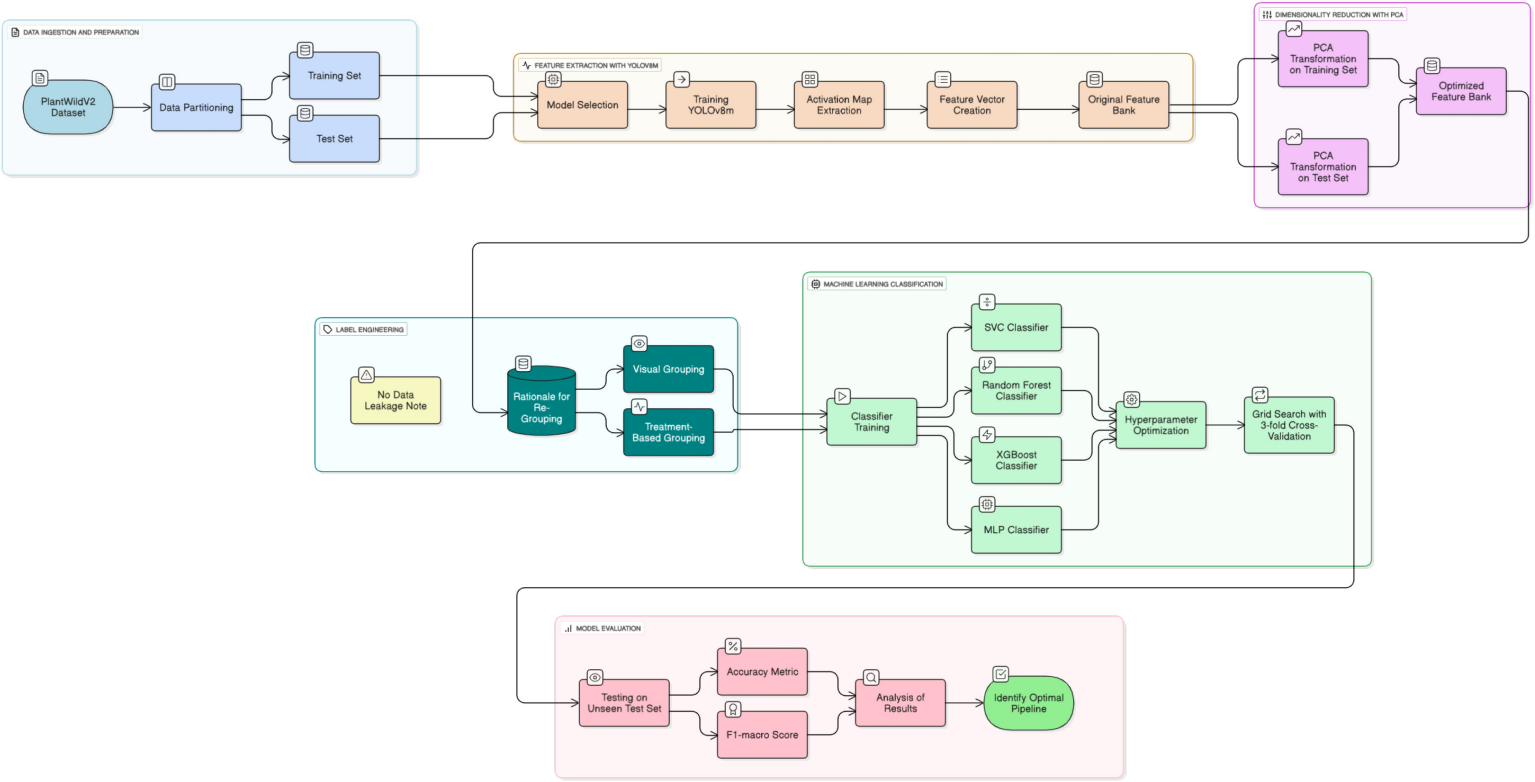
System architecture of the proposed two-stage classification pipeline.

We designed the methodology to evaluate a two-stage classification pipeline against an end-to-end baseline, with emphasis on the impact of the proposed label engineering strategy. **Figure 1** shows the workflow from data ingestion to evaluation, and **Algorithm 1** presents the formal steps.

Algorithm 1

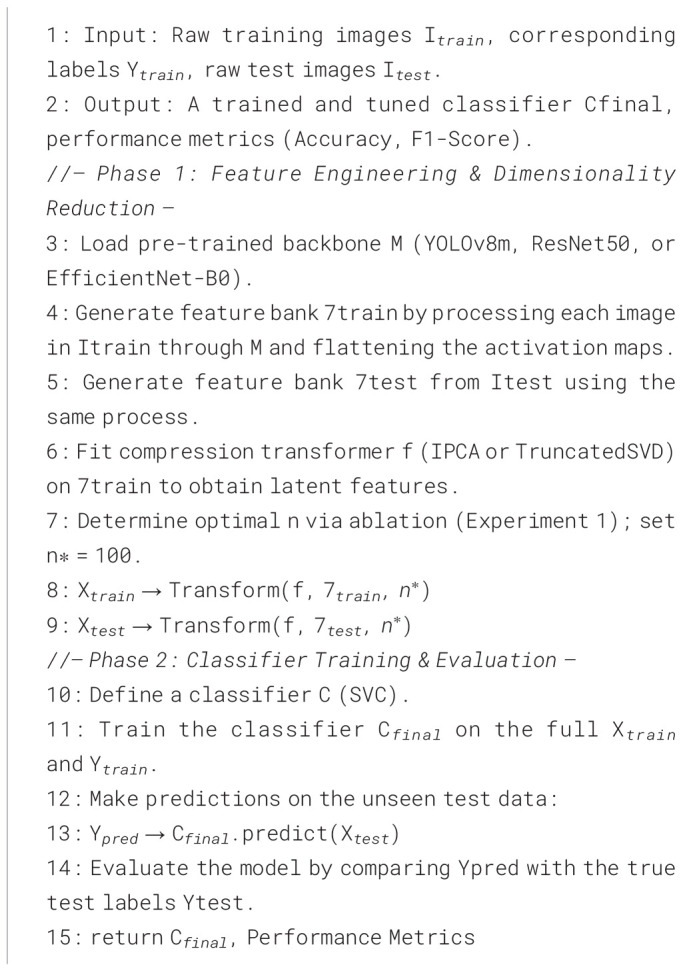

### 3.1 Dataset

**Figure 2:**
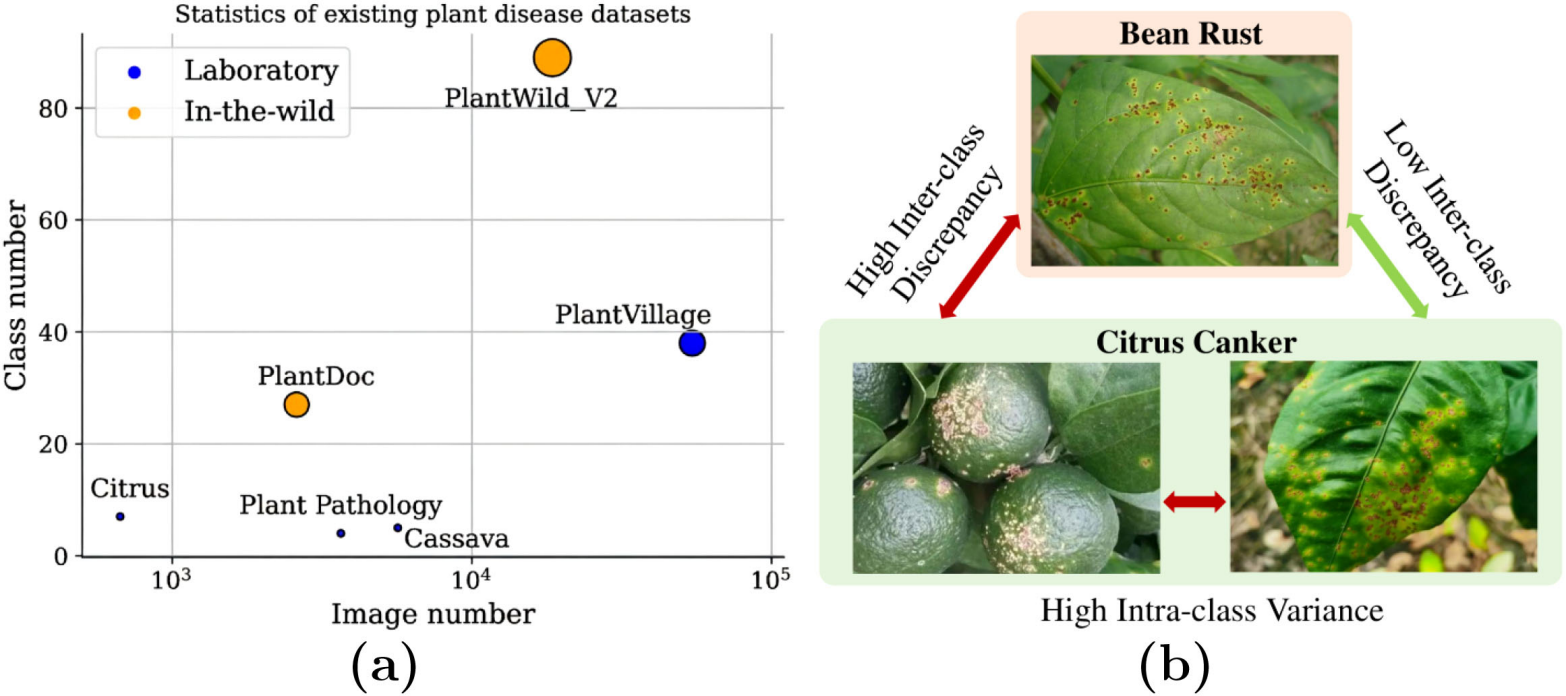
**(a)** The dataset with the highest volume of in-the-wild images. **(b)** Illustration of intra-class variance and inter-class similarity among plant disease images.

We use the PlantWildV2 dataset (Wei et al., 2024), a large-scale benchmark for in-the-wild plant disease recognition. It contains 11,349 images across 115 disease classes, with predefined training and test splits used without modification. **Table 1** include popular datasets and **Table 2** summarizes key statistics, and **Figure 2** illustrates intra-class variability and inter-class similarity. We selected this dataset to approximate real- world agricultural conditions and avoid the well-documented limitations of many lab-based datasets (Barbedo, 2016; Noyan, 2022).

**Table 1:** A summary of popular plant disease datasets.

Dataset	Plant	Size	Resolution	Setting
[20]	Pear	3,505	Multiple	Field
[35]	Coffee	4,407	2048×1024	Lab
[56]	Coffee	1,560	Multiple	Field
[76]	Apple	3,651	2048×1365	Field
[58]	Rice	120	2848×4288	Lab
[59]	Citrus	759	256×256	Lab
[28]	Multiple	54,309	Multiple	Lab

**Table 2:** PlantWildV2 dataset description.

	Train	Test
Number of Classes	115	115
Mean images/class	78.3	20.4
Total images	9,001	2,348

However, the accuracy on real-world datasets is marred by messy backgrounds as opposed to clean lab images with clean backgrounds. The problem that is being faced is that of bias in datasets, which can cause a false sense of accuracy. The problem has already been highlighted in PlantVillage. In one study (Noyan, 2022), trained a model on just eight background pixels on PlantVillage images and got 49.0% accuracy on the test set, much more than a random guess accuracy of 2.6%.

In order to validate the robustness of PlantWildV2, we tried out this 8-pixel background bias study on our dataset. We found that we got 12.87% accuracy on just backgrounds, as opposed to a random guess accuracy of 0.85%. There is still some remnant connection between field conditions and type of disease, but this is a significant reduction from 49% as seen in lab datasets. We rely on PlantWildV2 to avoid misleading signals and create a practical and robust model. 

### 3.2 Label engineering

**Figure 3:**
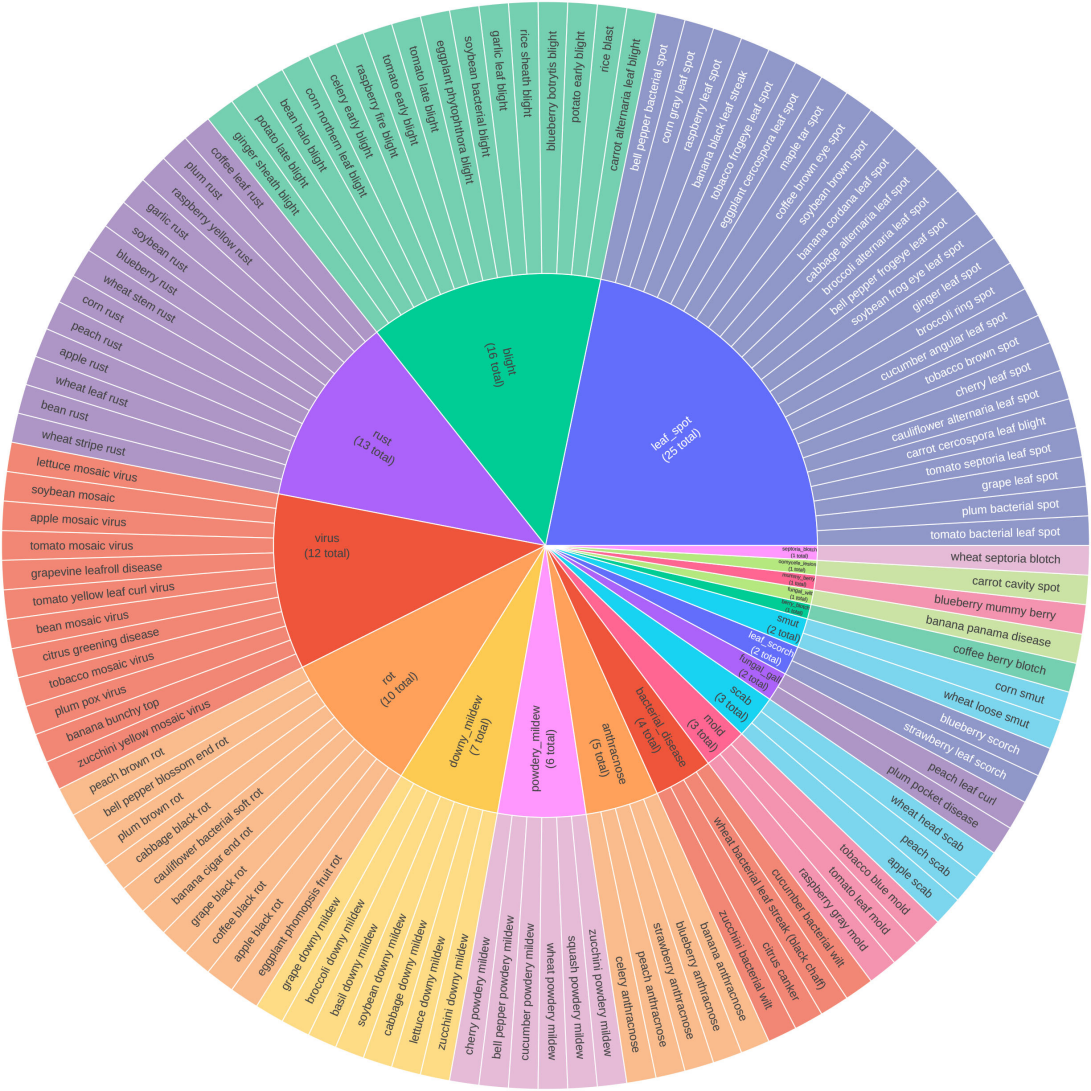
19-class visual-based grouping strategy.

**Figure 4:**
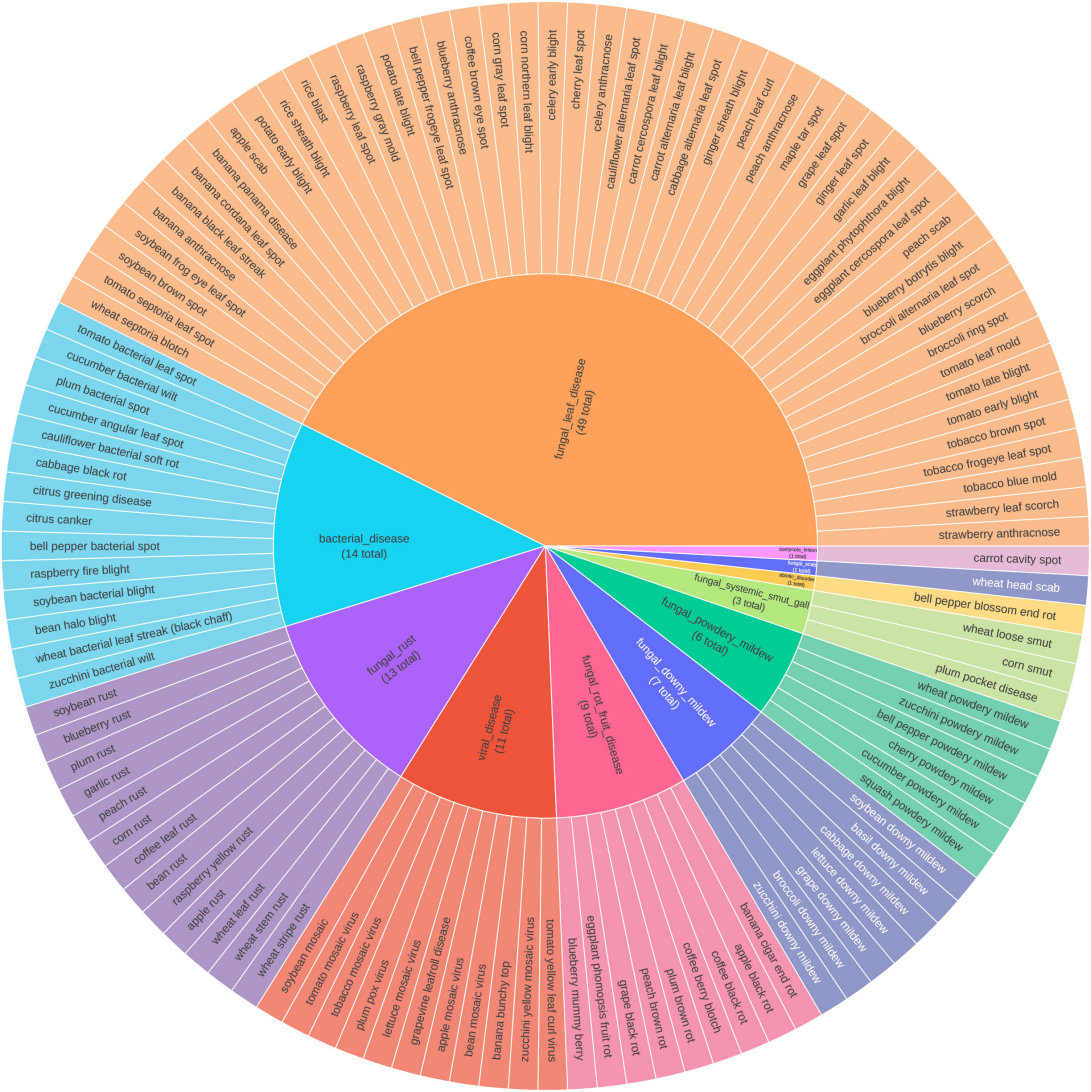
11-class treatment-based grouping strategy.

Given the high inter-class visual similarity among diseases, we evaluated two label consoli- dation strategies (**Figures 3**, **4**):

1. Visual Grouping: We mapped the 115 fine-grained classes to 19 super-classes based on shared visual nomenclature (e.g., grouping rust types under ‘Rust’).
2. Treatment-Based Grouping: We mapped the 115 classes to 11 actionable super-classes defined by pathogen type and management strategy (e.g., FungalRust, ViralDisease). A plant pathology expert reviewed this consolidation to ensure that merged categories correspond to consistent treatment protocols.

All experiments used the same images; only the target labels differed by grouping strategy.

### 3.3 Two-stage pipeline

The pipeline has three sequential stages—feature extraction, dimensionality reduction, and machine learning classification—designed to transform raw images into predictions (**Figure 1**). Decoupling feature extraction from classification supports lightweight retraining without re- optimizing the deep backbone, which is advantageous for scenarios requiring rapid model adap- tation and minimal computational cost.

Feature extraction. Following the established principle that pre-trained CNN features serve as powerful general-purpose representations (Sharif Razavian et al., 2014; Yosinski et al., 2014), we compared three state-of-the-art backbones used as frozen feature extractors:

- ResNet50 (He et al., 2016): A standard deep residual network widely used in image classification.
- EfficientNet-B0 (Tan and Le, 2019): A lightweight architecture optimized for mobile applications via compound scaling.
- YOLOv8m (Jocher et al., 2023): A modern object detection architecture adapted for feature extraction. We extracted activation maps from the final C2f block (Layer 8), selected as a trade-off between semantic depth and spatial resolution.

All backbones were pretrained on ImageNet (Russakovsky et al., 2015) (or COCO (Lin et al., 2014) in YOLO’s case) and fine-tuned on the PlantWildV2 training split. Features were extracted once and stored as a static bank.

Reduced Order Modelling via PCA/SVD. The high-dimensional feature maps ex- tracted from deep backbones contain significant redundancy (Jolliffe, 2002). We implemented Reduced Or- der Modelling (ROM) using two techniques: Incremental Principal Component Analysis (IPCA) (Ross et al., 2008) for memory-efficient streaming, and Truncated Singular Value Decomposition (SVD) (Halko et al., 2011). We compressed the feature space to a compact latent representation of n = 100 dimensions, as identified by our ablation study. The transformers were fitted only on the training set to prevent data leakage.

Classification. We trained a Support Vector Classifier (SVC) (Cortes and Vapnik, 1995; Vapnik, 1995) on the resulting low-dimensional representations. We used scikit-learn’s (Pedregosa et al., 2011) implementation with a radial basis function (RBF) kernel (Scholkopf and Smola, 2002) and class weighting to address imbalance (He and Garcia, 2009).

### 3.4 Experimental design

We conducted three sequential experiments to identify the optimal pipeline configuration. All experiments used the predefined PlantWildV2 training and test splits to ensure comparabil- ity with prior work on this benchmark. Hyperparameters for the SVC classifier (regularization parameter C and kernel coefficient γ) were selected via grid search with 3-fold stratified cross- validation on the training set only; the test set was held out and used exclusively for final evaluation.

Experiment 1: Optimal n_components Ablation. We aimed to identify the optimal number of principal components. We expected an intermediate dimensionality to retain signal while discarding noise. We trained SVC models with component counts ranging from 100 to 5,745 and identified n = 100 as the optimal trade-off point where overfitting was minimized.

Experiment 2: Backbone and Compression Comparison. Using the optimal dimen- sionality (n=100), we conducted a comprehensive comparison of three backbones (YOLOv8m, ResNet50, EfficientNet-B0) combined with two compression methods (IPCA, SVD). This re- sulted in six model configurations, evaluated on both the 19-class (Visual) and 11-class (Treatment- based) tasks. This experiment directly addresses the need for comparative validation against standard architectures.

Experiment 3: Final Model Validation. We decided to select our top performer, YOLOv8m with IPCA, for a thorough validation process, including per-class evaluation and a look into the confusion matrix.

## 4 Results

We will present our results in three sections. First, we will demonstrate our ablation study to verify our hypothesis about the optimal feature dimensionality. Then, we will compare our backbone approach to the YOLO-based one and verify our hypothesis about the superiority of the latter. In the third section, we will evaluate our winning model.

### 4.1 Optimal feature dimensionality: less is more

**Figure 5:**
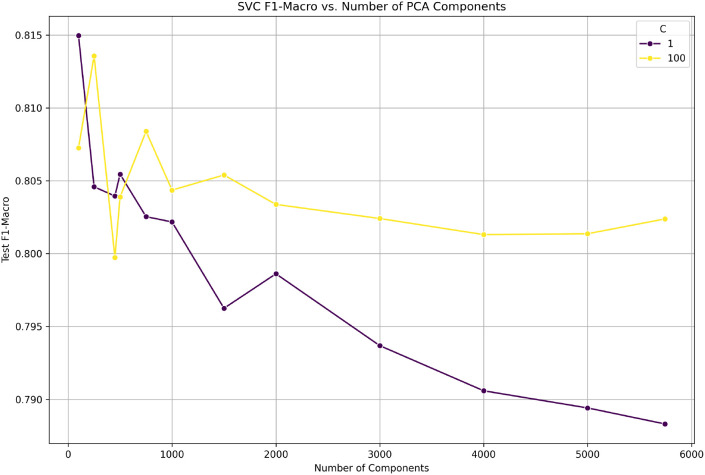
F1-macro as a function of the number of principal components. Performance peaks at n=100 and then degrades, indicating that strong dimensionality reduction regularizes the task.

In our initial experiment, we demonstrated that the performance of our model peaks when we use 100 principal components and then drops as we increase the dimensionality. This is quite unexpected, as one would assume that the more features we use, the better our model will perform. However, we believe that this is due to the strong regularization effect of feature com- pression, where we eliminate task-irrelevant background noise contained in high-dimensional feature representations. All subsequent results use As illustrated in **Figure 5**, the optimal trade-off is achieved at. Therefore, all subsequent experiments adopt, based on its ability to preserve the most informative features while maintaining strong generalization performance.

### 4.2 Backbone and method comparison

**Table 3** presents the performance of all tested configurations. The YOLOv8m backbone significantly outperformed ResNet50 and EfficientNet-B0 across all tasks. Specifically, the proposed YOLOv8m + IPCA pipeline achieved a test accuracy of 87.52%, surpassing the EfficientNet-B0 + IPCA pipeline (70.53%) by over 16 percentage points.

**Table 3:** Test performance of different backbones and compression methods (n=100). The YOLOv8m + IPCA pipeline achieves the highest accuracy, significantly outperforming standard architectures.

Labeling strategy	Backbone	Compression	ML model	Accuracy (%)	F1-macro	Training time (s)
	EfficientNet-B0	IPCA	SVC	68.23	0.705	33.5
	EfficientNet-B0	SVD	SVC	65.93	0.684	31.0
Visual Grouping (19 Classes)	ResNet50	IPCA	SVC	66.23	0.681	34.5
	ResNet50	SVD	SVC	59.63	0.618	32.8
	YOLOv8m (Champion)	IPCA	SVC	86.54	0.863	29.7
	YOLOv8m	SVD	SVC	86.03	0.854	26.4
	EfficientNet-B0	IPCA	SVC	70.53	0.706	37.8
	EfficientNet-B0	SVD	SVC	67.97	0.685	36.8
Treatment Grouping (11 Classes)	ResNet50	IPCA	SVC	68.27	0.679	40.4
	ResNet50	SVD	SVC	61.07	0.619	39.9
	YOLOv8m (Champion)	IPCA	SVC	87.52	0.882	30.8
	YOLOv8m	SVD	SVC	86.54	0.874	28.1

Interestingly, Incremental PCA (IPCA) yielded slightly better or comparable results to Truncated SVD, while offering the practical advantage of stream-processing large datasets without loading the entire feature bank into memory. The treatment-based (11-class) and visual-based (19-class) groupings showed identical high performance with the YOLO back- bone, suggesting its features are robust enough to capture the underlying pathology regardless of the specific label hierarchy.

### 4.3 In-depth analysis of the champion pipeline

We analyzed the champion model (YOLOv8 + IPCA(n=100) + SVC) on the 11-class treatment-based task.

#### 4.3.1 Per-class performance

The model demonstrates exceptional robustness, particularly on visually distinct classes. **Table 4** shows F1-scores exceeding 0.90 for fungal_powdery_mildew and abiotic_disorder. Even for challenging classes like fungal_rot_fruit_disease, the model maintains respectable performance.

**Table 4:** Per-class performance of the champion SVC model on the 11-class test set.

Class	Precision	Recall	F1-Score	Support
abiotic_disorder	0.85	1.00	0.92	23
bacterial_disease	0.83	0.80	0.81	320
fungal_downy_mildew	0.81	0.90	0.85	150
fungal_leaf_disease	0.90	0.85	0.87	835
fungal_powdery_mildew	0.95	0.94	0.95	188
fungal_rot_fruit_disease	0.78	0.81	0.79	152
fungal_rust	0.89	0.89	0.89	320
fungal_scab	0.86	0.98	0.92	64
fungal_systemic_smut_gall	0.79	0.86	0.82	96
oomycete_lesion	0.80	0.90	0.85	15
viral_disease	0.81	0.88	0.84	185
**Macro Avg**	**0.84**	**0.89**	**0.86**	**2,348**
**Weighted Avg**	**0.86**	**0.86**	**0.86**	**2,348**

Bold values indicate Macro Avg and Weighted Avg, the rest of rows are disease classes.

#### 4.3.2 Error analysis and model calibration

**Figure 6:**
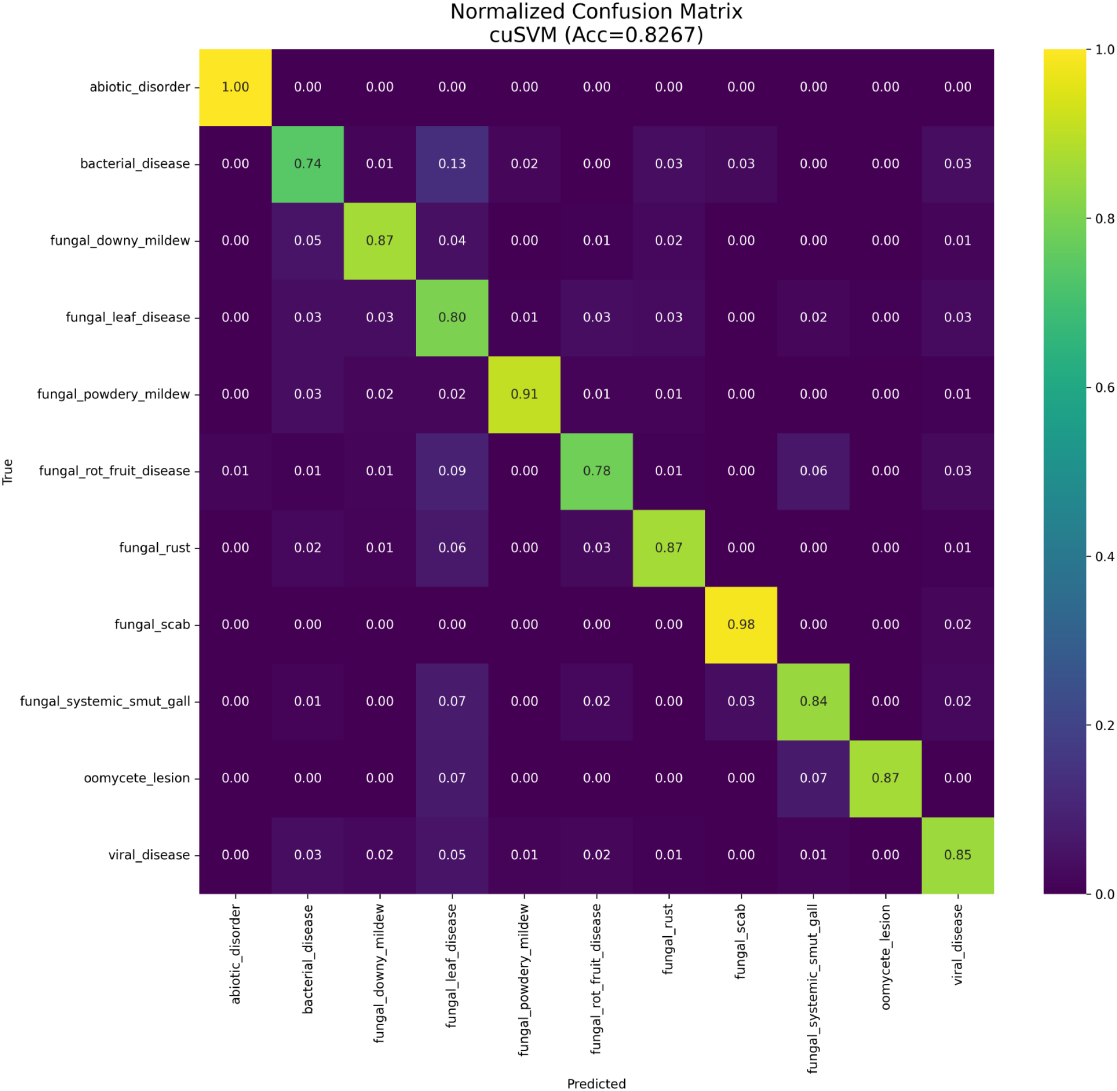
Normalized confusion matrix for the champion YOLO+IPCA model.

**Figure 7:**
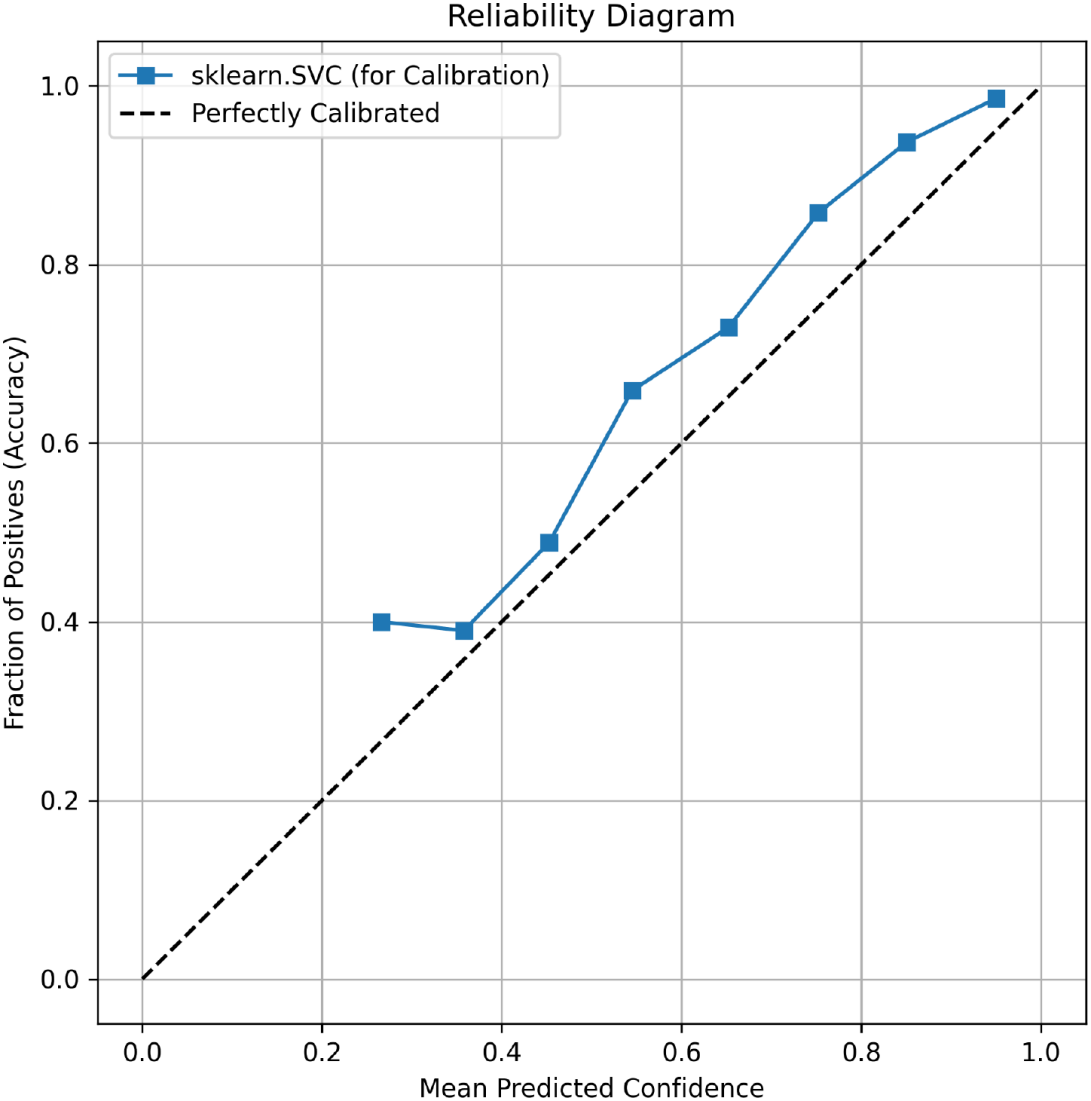
Reliability diagram for the champion model.

The confusion matrix (**Figure 6**) confirms low misclassification rates. The reliability dia- gram (**Figure 7**) indicates the model remains well-calibrated, a critical property for automated decision support systems in agriculture.

### 4.4 Comparison with high-resource end-to-end baselines

To evaluate the efficiency of our hybrid approach, we compare our approach with a typ- ical high-resource approach to image classification, which is the end-to-end fine-tuning of EfficientNet-B0. Theoretically, the end-to-end approach should always outperform all other approaches as the feature representations are optimized for the task.

However, as presented in **Table 5**, our approach outperforms the end-to-end approach with a test accuracy of 87.52% as opposed to the end-to-end approach with an accuracy of 82.50%. This indicates that our approach benefits from strong inductive biases of the YOLOv8 detection backbone, resulting in richer feature representations for detecting localized disease symptoms than the classification backbone.

**Table 5:** Comparison with high-resource baseline.

Model	Test acc.	Training time	Hardware	Retrai
EfficientNet-B0 (End-to-End)	82.50%	∼5.8 hours	GPU (A100)	
**Proposed Pipeline (YOLO+IPCA+SVC)**	**87.52%**	**30.8 seconds**	**CPU**	**Ne**

The proposed ROM framework outperforms the computation-heavy end-to-end model while drastically reducing training time.

Bold values indicate best pipeline.

Most importantly, our approach benefits from a significant runtime efficiency. The end- to-end approach requires heavy GPU resources to optimize the model via backpropagation, whereas our approach can retrain the classifier in just 30.8 seconds on a standard CPU. This translates to a whopping speedup of 670× over the end-to-end approach, making our approach suitable for dynamic agricultural environments where diseases may need to be frequently up- dated.

### 4.5 Fairness of backbone comparison

A potential confound in our main results is that YOLOv8m was pre-trained on COCO (de- tection), whereas ResNet50 and EfficientNet-B0 were pre-trained on ImageNet (classification). To control for this, we fine-tuned ResNet50 and EfficientNet-B0 on the PlantWildV2 training split for 15 epochs using the Adam optimizer (lr=10−4), then re-extracted features and re-ran our pipeline. As shown in **Table 6**, even with target-domain fine-tuning, the YOLOv8m back- bone (without any additional fine-tuning) still outperforms both baselines, confirming that the advantage is structural rather than an artifact of pre-training data.

**Table 6:** Fairness comparison: YOLOv8m (COCO pre-trained, not fine-tuned) vs. classification backbones fine- tun e d on PlantWildV2 for 15 epochs.

Pipeline	Test acc. (%)	F1-macro	Fine-tuned?
EfficientNet-B0 + IPCA + SVC	76.87	0.76	Yes (15 epochs)
ResNet50 + IPCA + SVC	85.05	0.843	Yes (15 epochs)
**YOLOv8m + IPCA + SVC**	**87.52**	**0.882**	No

The detection backbone retains a clear advantage.

Bold values indicate best pipeline.

### 4.6 Statistical significance: McNemar’s test

To formally validate the performance differences observed in **Table 6**, we conducted pairwise McNemar’s tests (McNemar, 1947) on the per-sample predictions of each backbone’s SVC classifier over the 2,348-sample test set. McNemar’s test evaluates whether the disagreements between two classifiers are statistically significant by constructing a 2 × 2 contingency table of concordant and discordant predictions. We applied the continuity-corrected variant with one degree of freedom.

**Figure 8:**
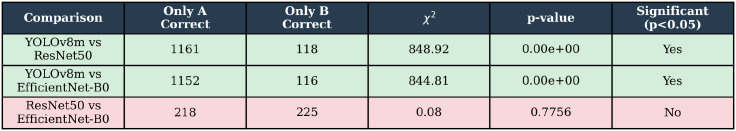
McNemar’s test: pairwise statistical comparison of backbone classifiers on the 2,348-sample test set. Green rows indicate statistically significant differences (p *<* 0.05).

**Figure 8** presents the results. The YOLOv8m pipeline produces statistically significant improvements over both ResNet50 (χ2 = 848.92, p < 10−6) and EfficientNet-B0 (χ2 = 844.81, p < 10−6). In both comparisons, YOLOv8m correctly classifies approximately 10× more sam- ples that the alternative misclassifies than the reverse, confirming that the accuracy gap is not attributable to chance. By contrast, ResNet50 and EfficientNet-B0 show no significant differ- ence (χ2 = 0.08, p = 0.776), indicating that the two classification backbones are statistically interchangeable under our pipeline.

### 4.7 Feature space visualization

**Figure 9:**
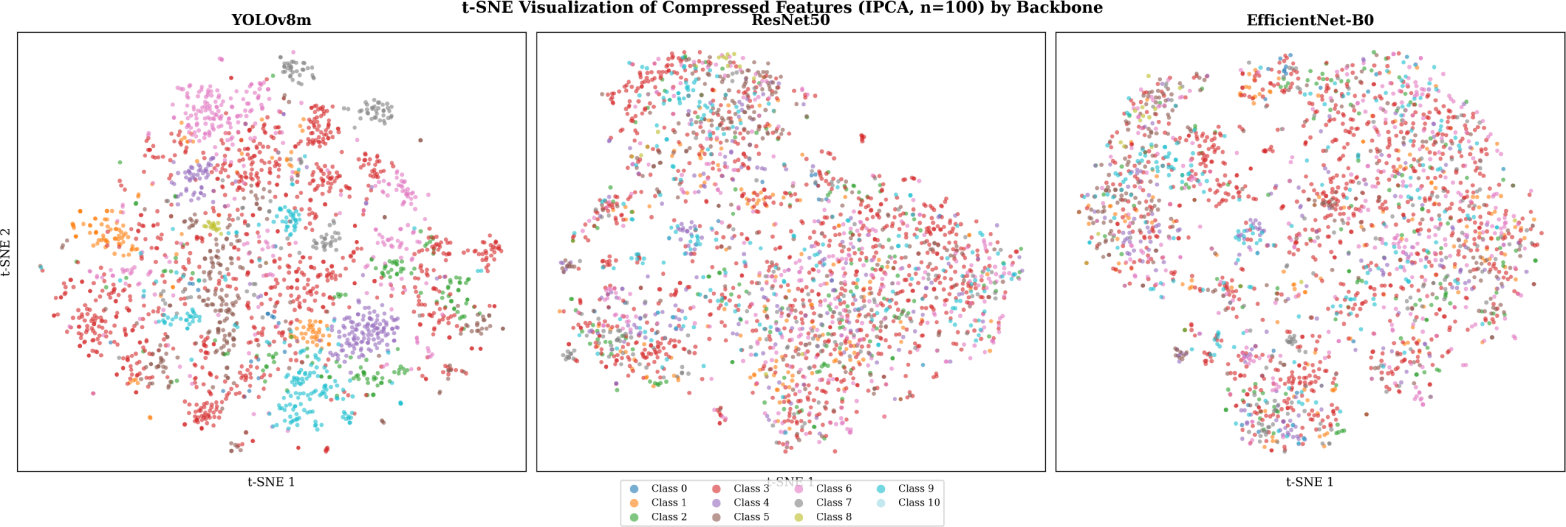
t-SNE visualization of IPCA-compressed features (*n* = 100) across backbones. YOLOv8m produces tighter, better-separated class clusters compared to ResNet50 and EfficientNet-B0.

To provide qualitative evidence for the superior discriminability of YOLOv8m features, we computed t-SNE embeddings (van der Maaten and Hinton, 2008) of the IPCA-compressed test features (n = 100) for all three backbones. **Figure 9** shows the resulting 2D projections colored by treatment-based class label.

The YOLOv8m features form visually tighter, better-separated clusters, consistent with its higher classification accuracy. ResNet50 and EfficientNet-B0 produce more diffuse and overlapping clusters, particularly for visually similar classes such as fungal leaf diseases and bacterial diseases. This visualization corroborates the quantitative findings in **Table 6** and **Figure 8**: the detection backbone learns more discriminative representations for localized disease patterns.

### 4.8 Computational efficiency analysis

To validate our assertion of the viability of edge deployment, the specifics of the parameters, footprint, and CPU inference latency per image at each component of the system are presented in **Table 7**, with benchmarks performed on an x86_64 CPU without GPU, averaging 50 runs with 5 warm-up iterations.

**Table 7:** Per-component computational profile of the proposed pipeline (CPU inference).

Component	Params (M)	Size (MB)	Latency (ms)	Output dim.
Ext: ResNet50	23.51	89.68	62.8 ± 1.8	2,048
Ext: EfficientNet-B0	4.01	15.29	25.8 ± 1.4	1,280
Ext: YOLOv8m	25.89	60.73	159.3 ± 11.0	192,000
Cls: IPCA Transform	0.21	0.79	0.42	100
Cls: SVC Inference	0.13	0.5	0.55	11
Classification Head Total	0.34	1.29	0.97	—

The lightweight classification head (IPCA + SVC) requires only 1.29 MB and <1 ms per image.

The IPCA + SVC classifier has a footprint of merely 1.29 MB, with an inference latency of less than 1.0 ms per image, demonstrating the efficiency of the downstream classifier for edge deployment. The major latency contribution is the initial feature extraction step. The YOLOv8m model is slower than the EfficientNet-B0 model by a wide margin of 159.3 ms vs. 25.8 ms, primarily due to the input size of 640×640, as opposed to the 224×224 input size of the EfficientNet-B0 model. However, this feature extraction step is performed only once per image, allowing the feature map to be cached, suitable for batch processing in an agricultural monitoring system.

## 5 Discussion

The experiments provide guidance for building accurate and efficient classifiers on in-the- wild plant disease data and clarify interactions among feature source, label design, and classifier choice. We discuss the key findings in relation to the broader literature.

### 5.1 YOLOv8 as a feature extractor for phytopathology

We show that a YOLOv8m detection backbone (Jocher et al., 2023), originally optimized for localization, can serve as a strong feature extractor for fine-grained disease classification. Our best pipeline (YOLOv8 features + IPCA + SVC) achieved a test accuracy of 87.52%, surpassing a fine- tuned EfficientNet-B0 baseline (82.5%) under the same data split and evaluation protocol. This finding is consistent with the broader observation that CNN features trained on large-scale tasks transfer effectively to domain-specific problems (Sharif Razavian et al., 2014; Yosinski et al., 2014). YOLOv8 produces spatially rich activation maps that remain discriminative after flattening and PCA compression, supporting the hypothesis that detection backbones—trained to localize objects within complex scenes— are well suited to capturing localized lesion patterns in field imagery (Wei et al., 2021).

This advantage is confirmed by both statistical testing and feature visualization. Pairwise McNemar’s tests (Section 4.6) demonstrate that YOLOv8m’s superiority over ResNet50 and EfficientNet-B0 is statistically significant (p < 10−6), while the two classification backbones are statistically interchangeable (p = 0.776). The t-SNE visualization (Section 4.7) provides complementary qualitative evidence: YOLOv8m features form tighter, better-separated class clusters in the compressed latent space, indicating that the detection backbone preserves more discriminative structure through the dimensionality reduction stage.

### 5.2 The impact of label engineering

The most significant improvements were obtained by rethinking the label space. Moving from the 19-class Visual Grouping to the 11-class Treatment-Based Grouping increased the accuracy and macro-F1 of all classifiers. This is in line with the assumption that high inter- class similarity is a major bottleneck (Wei et al., 2021).

### 5.3 Limitations

We tested the model on a single dataset, namely the PlantWildV2 benchmark (Wei et al., 2024). The dataset was selected due to the realistic conditions of the data, as well as its size, as it is the largest publicly available field-condition benchmark dataset for plant disease recognition.

### 5.4 Model generalization and practical deployment

From our findings, tree-based ensembles such as RandomForest (Breiman, 2001) and XGBoost (Chen and Guestrin, 2016) al- most saturated the training data but had significant generalization gaps (Sahin, 2020). In contrast, SVC (Cortes and Vapnik, 1995) had the highest accuracy on the test data with the smallest gap, implying that the YOLOv8+PCA feature space works well with the maximum margin principle (Vapnik, 1995). The two-stage approach provides two advantages: high accuracy on the test data with good general- ization ability, and a modular structure for quick retraining and efficiency, which is important when considering scenarios with tight memory and computational constraints (Bhagat et al., 2024; Nyakuri et al., 2025). This structure will prove beneficial when implementing the model with IoT-based monitoring sys- tems, as the model will need to be frequently retrained to accommodate the appearance of new strains of diseases (Liakos et al., 2018).

## 6 Conclusion

In this paper, we explored plant disease classification in the wild on the PlantWildV2 dataset with a two-stage approach, separating deep feature extraction from classification. The YOLOv8m model used as a feature extractor proved to be spatially aware, suitable for plant symptoms.

We demonstrated the importance of label engineering in plant disease classification. The aggregation of the 115 classes into 11 classes based on treatment types improved accuracy and macro-F1 across all models. This highlights label engineering as one of the most important steps in optimizing plant disease classification models.

We achieved an accuracy of 87.52% and a macro-F1 of 0.882 with our optimal pipeline, YOLOv8+PCA+SVC. Although this is only slightly higher than our baseline EfficientNet-B0 model with a fine-tuning accuracy of 82.5%, the two-stage approach provides advantages in retraining the model and efficiency, making it suitable for scenarios with tight constraints.

This approach will be taken further in three ways in future work. First, we will evaluate different feature extractors, such as Vision Transformers (Dosovitskiy et al., 2021) and small YOLO architectures, by exploiting recent advances in hybrid architectures (Salman et al., 2025) to improve accuracy-efficiency trade- offs. Second, we will explore hierarchical classification approaches to provide treatment-level as well as disease identification. This will involve exploring multi-output learning strategies (Fenu and Malloci, 2021). Finally, we will explore domain adaptation approaches (Wu et al., 2023) to understand the generalization of our pipeline to other crop species and geographic locations. This will help to provide robust and accurate diagnostic tools for sustainable agriculture.

## Data Availability

The original contributions presented in the study are included in the article/supplementary material. Further inquiries can be directed to the corresponding author.
